# Supplementary material for: Polymorphism Crystal Structure Prediction with Adaptive Space Group Diversity Control
Source: Adv Sci (Weinh). 2025 Sep 26;12(47):e10792. doi: 10.1002/advs.202510792 (PMC12713066; doi:10.1002/advs.202510792)
Supplement: Supplementary file 1 — Supporting Information [file ADVS-12-e10792-s001.pdf]

Supporting Information: Polymorphism Crystal Structure Prediction with Adaptive Space Group Diversity Control

Sadman Sadeed Omee,[†] Lai Wei,[†] Sourin Dey,[†] and Jianjun Hu[*,†,‡]

†*Department of Computer Science and Engineering, University of South Carolina, Columbia, SC, 29201*

‡*Corresponding author*

E-mail: jianjunh@cse.sc.edu

Contents

S1 Supplementary Notes **S-2**

S1.1 Pseudo-Code of ParetoCSP2 . . . . S-2

S1.2 Experimental Setup S-3

S1.3 Evaluation Metrics S-3

S2 Supplementary Figures **S-6**

S3 Supplementary Tables **S-15**

References **S-33**

S1 Supplementary Notes

S1.1 Pseudo-Code of ParetoCSP2

We present the pseudo-code of our ParetoCSP2 algorithm here.

Algorithm 1: ParetoCSP2: Polymorphism crystal structure prediction with adaptive space group diversity control

Input: Chemical composition C
Output: Best structure(s) with lowest energy and high space group diversity

Initialization:
Generate initial population $\mathcal{P}_0$ from C using `PyXtal`;
foreach individual $i \in \mathcal{P}_0$ **do**
    Set $a(i) = a(i) + 1$
Set generation count $g = 0$;
while $g < \mathcal{G}$ **do**
    Energy & Diversity Evaluation:
    foreach individual $i \in \mathcal{P}_g$ **do**
        Get the structure s by decoding i;
        Predict energy per atom $E(s)$ using an ML IAP (e.g., M3GNet);
        Set the shared space group count $ssgc(s)$ by assigning the total number of structures sharing the same space group; Encode the structure s back to the individual vector i.
    Fitness Evaluation:
    foreach individual $i \in \mathcal{P}_g$ **do**
        Compute fitness $F(i)$ based on $E(i)$, $a(i)$, and $ssgc(i)$;
    Parent Selection:
    Perform Pareto tournament selection to choose parents for mating;
    Genetic Operations:
    foreach selected parent pair **do**
        Apply crossover → offspring inherits $\max(a_{parent1}, a_{parent2})$;
        Apply mutation → offspring inherits a_{parent};
    Population Update:
    Add new offspring to form $\mathcal{P}_{g+1}$;
    Space Group Tracking:
    Store best j structures per k distinct space groups based on the energy for polymorphism analysis;
    Age update:
    foreach individual $i \in \mathcal{P}_{g+1}$ **do**
        Set $a(i) = a(i) + 1$
    Shallow relaxation: foreach individual $i \in \mathcal{P}_g$ **do**
        Get the structure s by decoding i;
        Shallow relax s using an ML IAP (e.g., M3GNet) Encode the structure s back to the individual vector i.
    Increment Generation:
    $g \leftarrow g + 1$;
Post-processing:
Perform deep relaxation on non-dominated structures;
Apply structure symmetrization using `Pymatgen`;
return *Best relaxed structure (non-polymorphic) or all jk relaxed structures (polymorphic).*

S1.2 Experimental Setup

For the implementation of GN-OA,[S1] we utilized the `PySwarms` package.[S2] We set the cognitive and social parameters to 0.5 and the inertia weight to 0.8 for all the experiments. Both ParetoCSP[S3] and ParetoCSP2 were implemented using the multi-objective optimization framework `Pymoo`.[S4]

Although additional parameter tuning may result in much more improved performance for ParetoCSP2, it was not explored in depth in this work. We carried out the experiments for ParetoCSP2 in two different settings. For NSGA-III,[S5,S6] experiments were conducted with a crossover probability of 0.8 and a mutation probability of 0.01. In most cases, a population size of 100 was used. In another setting, we used a crossover probability of 0.9 and a mutation probability of 0.1, and a population size of 200. We selected the Simulated Binary Crossover[S7] as the crossover operator and the Polynomial Mutation[S7] as the mutation operator, where the Polynomial Mutation follows the same probability distribution as the Simulated Binary Crossover. For AFPO,[S8,S9] the tournament size for the Pareto tournament selection was set at 2. The `PyXtal`[S10] library was used to generate the initial population set in ParetoCSP2.

The M3GNet universal IAP[S11] was used for structure relaxation in most cases, and CHGNet[S12] for others. A better IAP such as SevenNet[S13] or eSEN[S14] can be used to improve performance. We used a step size of only 10 for the shallow relaxation of structures after each generation and a step size of 500 for the final deep structure relaxations. The Python Materials Genomics (`Pymatgen`)[S15] library was used for structure symmetrization. We kept track of a total of 10 different space group structures for the polymorphism prediction experiments and kept the number of structures to track with the same space group to three. All three algorithms were run for a maximum of 500 generations.

Crystal structure visualizations were performed using the `VESTA` software package.[S16] All experiments were carried out in the Hyperion Cluster of the University of South Carolina using Nvidia Tesla V100 GPUs. The experiments took a time ranging from a few hours to 3-5 days depending on the complexity of the crystal's elemental composition and possible Wyckoff combinations.

S1.3 Evaluation Metrics

The evaluation metrics used in this study described in detail are described here. For more details, the readers are referred to the work of Wei et al.[S17]

1. **Energy distance:** The energy distance (ED) calculates the difference between a predicted structure and its ground-truth structure. It is computed as the absolute difference in their total energy per atom, typically using machine-learned potentials like M3GNet. Although a smaller ED often indicates a structure closer to the ground state in stability, it does not guarantee structural similarity. For example, graphite and diamond have small energy differences but distinct structures. So, it should be used alongside geometric metrics. ED can be calculated using the following equation:

$$\mathrm{ED} = |E_P - E_G| \tag{1}$$

In Eq. 1, E_P and E_G denote the energy per atom of the predicted structure and the ground truth structure, respectively.

2. **Sinkhorn distance:** The Sinkhorn distance (SD) is a regularized version of the Earth Mover's distance[S18] used to compare atomic point clouds between structures. It models the optimal transport of mass between atoms of two crystal structures, allowing for a smooth and differentiable way to quantify their similarity. SD is robust to small perturbations and does not depend on atom ordering or simulation cell constraints, which makes it useful for comparing structures with minor geometric changes. SD can be calculated using the following equation:

$$\mathrm{SD}(P, G) = \frac{1}{\epsilon}\left(\sum_{i,j} T_{i,j} \log\left(\frac{T_{i,j}}{u_i v_j}\right) - \log C\right) \tag{2}$$

In Eq. 2, $P = \{p_1, p_2, \ldots, p_n\}$ and $G = \{g_1, g_2, \ldots, g_n\}$ denote the atomic sites of the predicted structure(P) and the ground-truth structure(G), respectively, $T_{i,j}$ denotes the optimal transport plan from point i in P to point j in G, u_i, v_j denote marginal distributions over P and G, ϵn denotes the entropic regularization parameter, and C denotes a normalization constant.

3. **Chamfer distance:** The Chamfer distance (CD) measures the distance between two structures by comparing each point in one structure to the nearest point in the other. It does this for both directions and then averages the squared distances. CD provides a measure of how well the atomic positions of one structure align with those of another. Although CD is computationally fast, it may be less sensitive to structural details, such as bonding patterns or atom types. CD can be computed by the following equation:

$$\mathrm{CD}(P, G) = \frac{1}{|P|}\sum_{p\in P}\min_{g\in G}\|p-g\|^2 + \frac{1}{|G|}\sum_{g\in G}\min_{p\in P}\|g-p\|^2 \tag{3}$$

In Eq. 3, P, G denote the sets of atomic sites in the predicted and ground truth structures, respectively, and $\|\cdot\|^2$ denotes the squared Euclidean distance.

4. **Hausdorff distance:** The Hausdorff distance (HD) measures the greatest distance between any point in one set of atomic sites and the nearest point in the other set. HD highlights the largest deviation between two structures. Unlike CD, which averages distances, HD focuses on the largest difference, making it more sensitive to outliers. HD is symmetric and is particularly useful for detecting significant structural differences between two structures. HD can be determined by the following equation:

$$\mathrm{HD}(P, G) = \max\left\{\sup_{p\in P}\inf_{g\in G}\|p-g\|, \sup_{g\in G}\inf_{p\in P}\|g-p\|\right\} \tag{4}$$

In Eq. 4, P, G denote the sets of atomic sites in the predicted and ground truth structures, respectively, sup denotes the maximum over all elements in the set, inf denotes the minimum distance to the other set, and $\|\cdot\|$ denotes any distance measure.

5. **Fingerprint distance:** The fingerprint distance (FP) is based on comparing the CrystalNN[S19] fingerprints, which capture the local coordination environment around each atom in a structure. Each fingerprint is represented as a vector that encodes coordination numbers and geometric configurations. An FP of zero indicates identical coordination environments, which makes this metric particularly effective for identifying chemically significant structural differences. FP can be measured by the following equation:

$$\text{FP} = \|\mathbf{f}_A - \mathbf{f}_B\|_2 \tag{5}$$

In Eq. 5, $\mathbf{f}_P, \mathbf{f}_G$ denote the feature (fingerprint) vectors of the predicted and ground-truth structures, respectively, and $\| \cdot \|_2$ denotes the L2 norm.

S2 Supplementary Figures

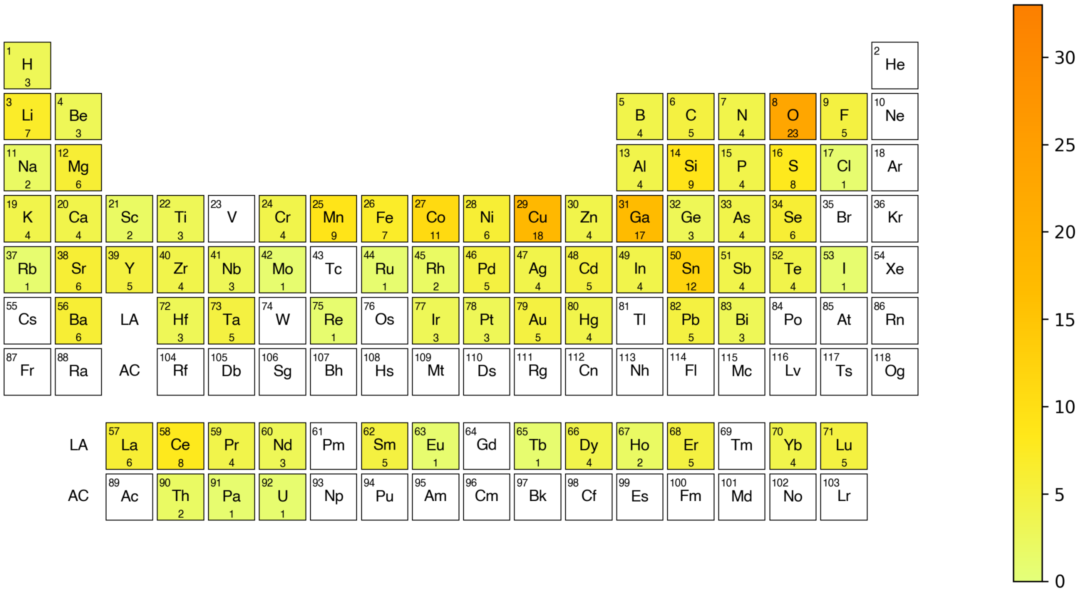

(a) ParetoCSP2

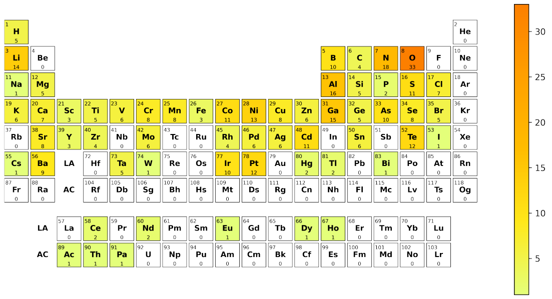

(b) ParetoCSP

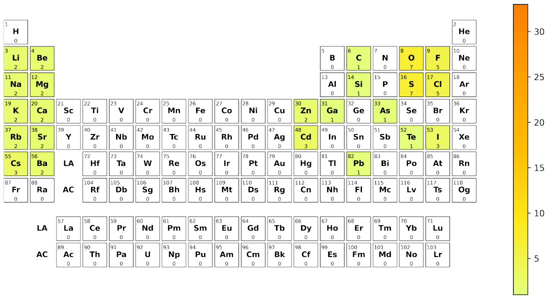

(c) GN-OA

Figure S1: Count of each element used in the benchmark set. (a), (b), and (C) show the element count for ParetoCSP2, ParetoCSP and GN-OA, respectively, while the color bars indicate the range of count and color scheme for that range. Compared to GN-OA and ParetoCSP's benchmark set, our chosen set is more diverse and covers a large portion of the periodic table.

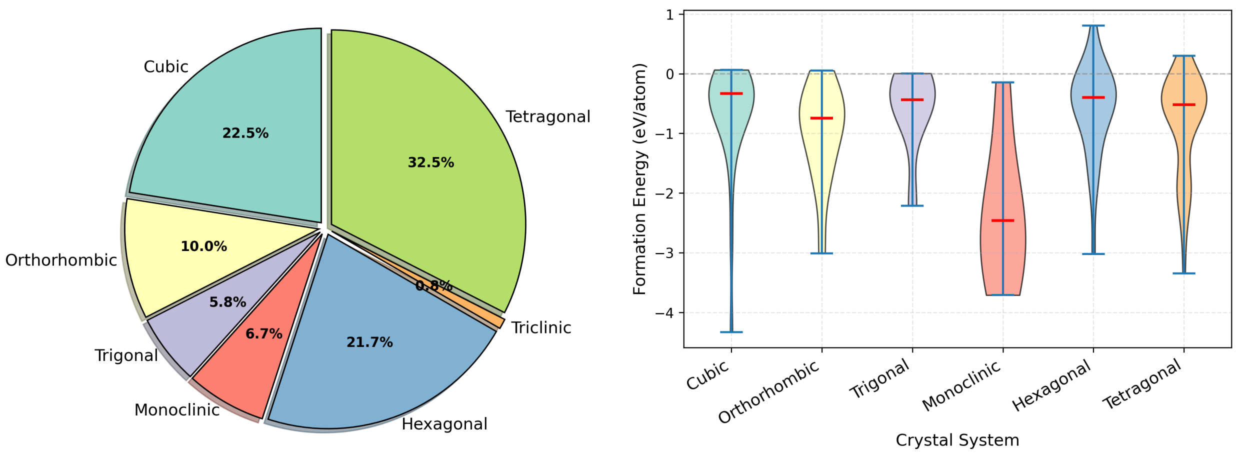

Figure S2: Distribution of crystal systems in the dataset and the corresponding formation energies. The pie chart (left) illustrates the distribution of crystal systems in the benchmark dataset, showing a majority of cubic, tetragonal, and hexagonal structures. The violin plot (right) shows the formation energy (eV/atom) distribution for each crystal system. Violin plots show that formation energies vary widely by crystal system. The monoclinic system exhibit deeper energy wells, indicating higher structural complexity and stability potential. This further justifies the need for high diversity in CSP algorithms to capture low-energy structures in complex symmetry classes as ParetoCSP2 underperformed on this particular system despite its overall superior prediction performance. Only one crystal ($RbLi_3S_2O_9$) belongs to the triclinic system which is not explicitly shown in the figure.

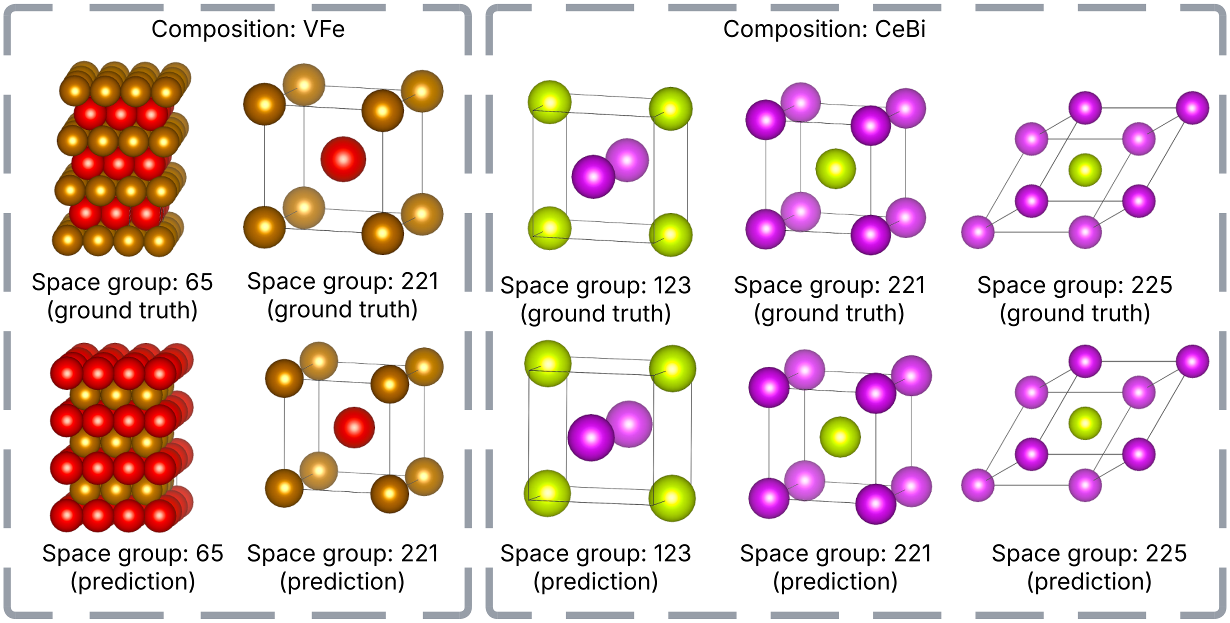

Figure S3: Sample polymorphism prediction of ParetoCSP2 For each chemical composition, the ground truth space groups are listed alongside the predicted space groups from the algorithm.

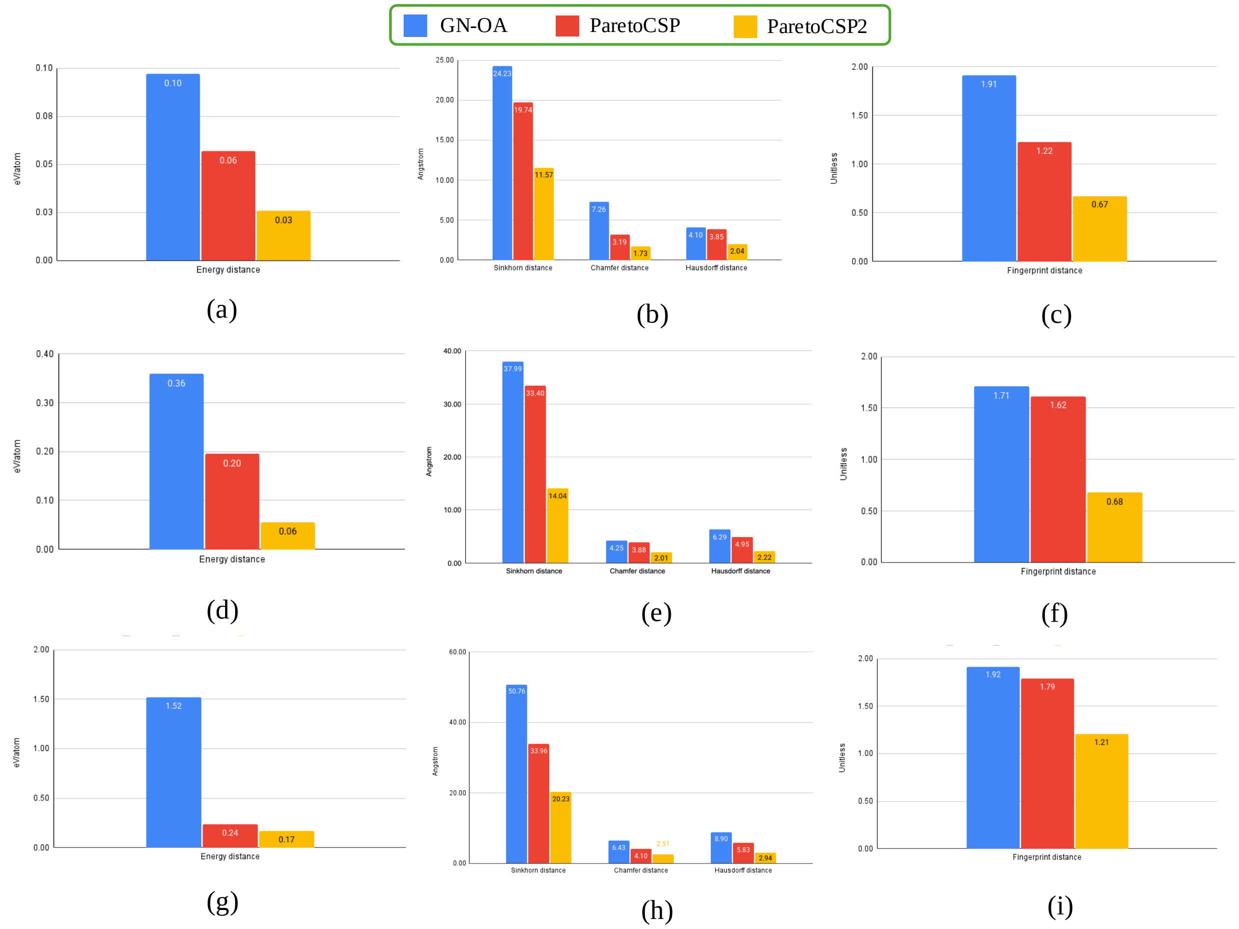

Figure S4: Performance metrics' comparison of ParetoCSP2 vs ParetoCSP and GN-OA for binary, ternary, and quaternary benchmark set crystals separately. The plots are divided by type of unit for better comparison: (a), (d), (e) represents ED for binary, ternary, and quaternary crystals, respectively, (b), (e), (h) represents SD, CD, and HD, for binary, ternary, and quaternary crystals, respectively, and (c), (f), (i) represents FP for binary, ternary, and quaternary crystals, respectively. ParetoCSP2 achieved performance improvements over the other two algorithms ranging from 41.37% to 76.17% for binary crystals, 48.20% to 83.33% for ternary crystals, and 29.17% to 88.82% for quaternary crystals, demonstrating its effectiveness in general CSP tasks for different genres of crystals.

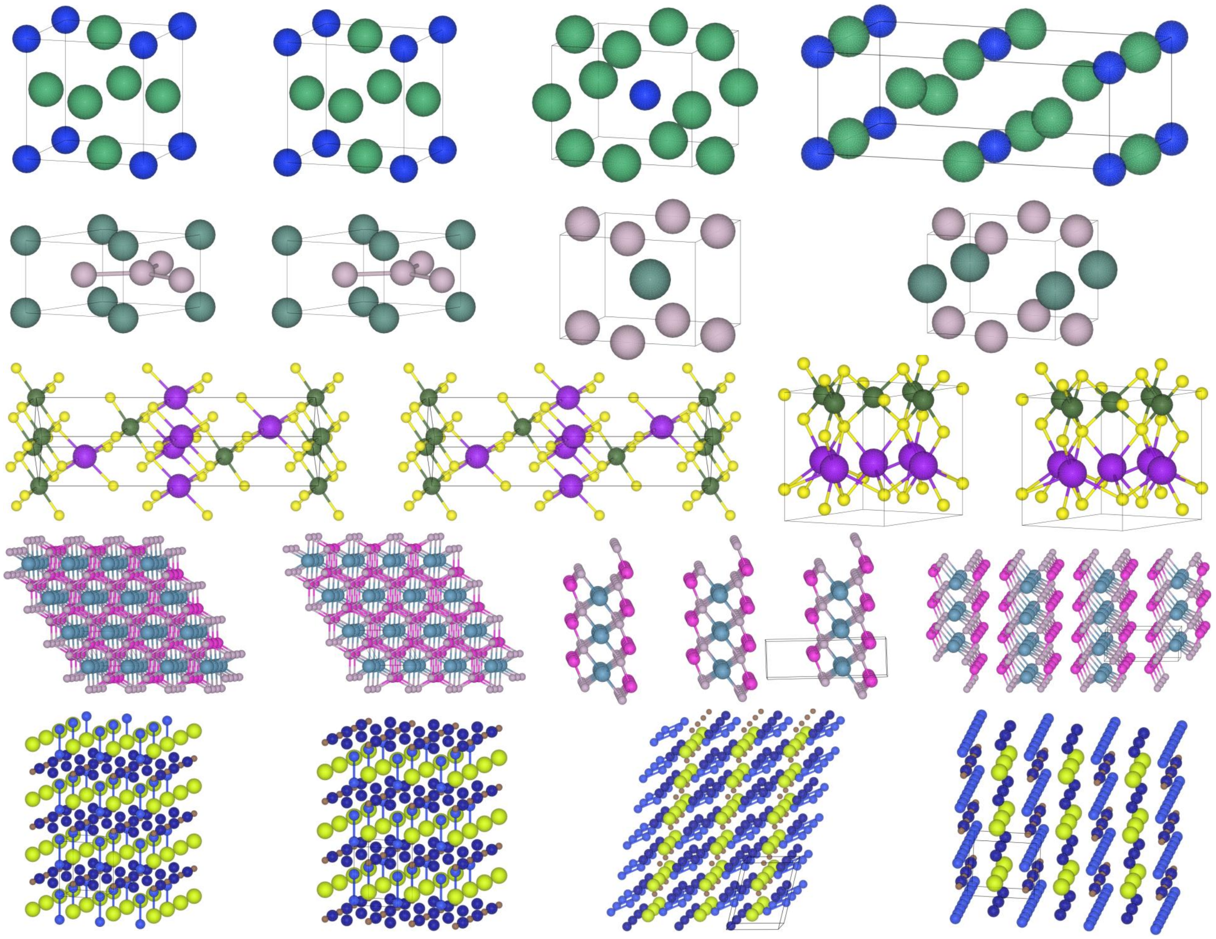

Figure S5: Sample successful structure predictions by ParetoCSP2 compared to ParetoCSP and GN-OA. Each row presents the ground truth structure, followed by its corresponding predicted structures from ParetoCSP2, ParetoCSP, and GN-OA, respectively. The selected examples - Nb_3Si, YHg_2, $KErS_2$, $Ca(CdP)_2$, and $CeCr_2Si_2C$ are ordered from top to bottom in decreasing crystallographic symmetry. All structures were visualized using VESTA. For better visualization, the fractional coordinate ranges for all axes were set to a maximum of 3 for $Ca(CdP)_2$ and $CeCr_2Si_2C$, and 1 for all other structures. The ball-and-stick style were used for presenting atoms and bonds. The lack of diverse space group exploration method in ParetoCSP and GN-OA resulted in unsuccessful predictions, even for high-symmetry structures such as Nb_3Si.

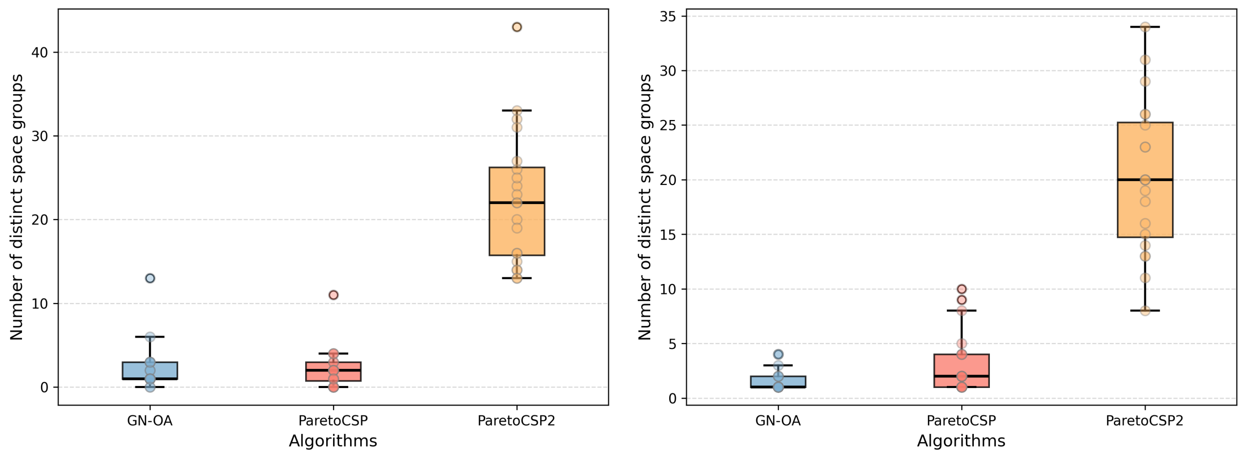

(a) (b)

Figure S6: Space group diversity comparison at early and final stages. Box plots show the number of distinct space groups generated by each algorithm after (a) 50 generations and (b) after 500 generations (right). ParetoCSP2 significantly outperforms GN-OA and ParetoCSP in both stages, achieving notably higher space group diversity. This highlights ParetoCSP2's effectiveness in exploring a broader symmetry space early to avoid premature local minima and sustaining diversity throughout the evolutionary process—critical for polymorph discovery.

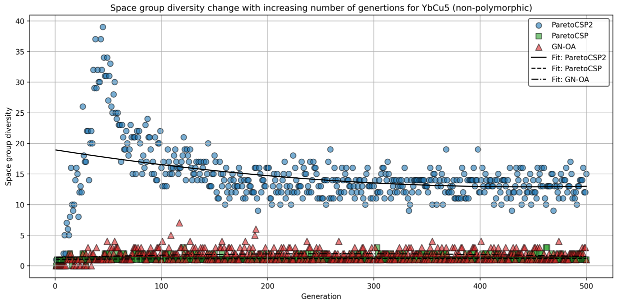

(a) $YbCu_5$

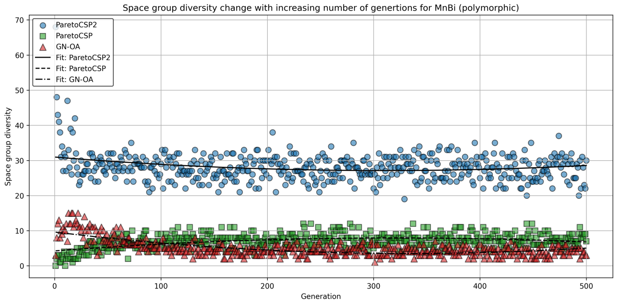

(b) MnBi

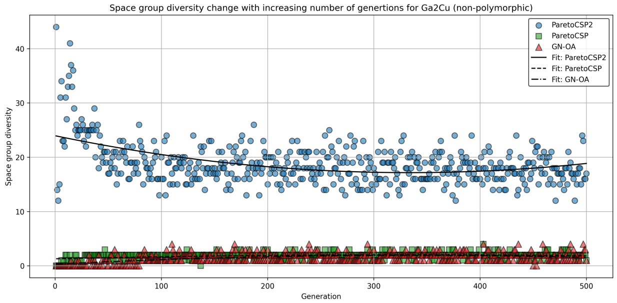

(c) Ga_2Cu

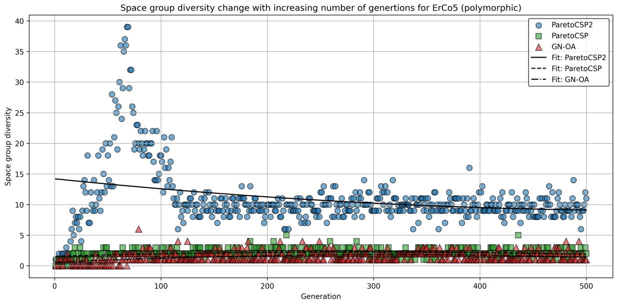

(d) $ErCo_5$

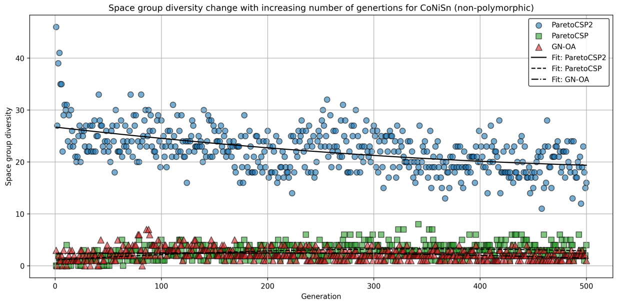

(e) CoNiSn

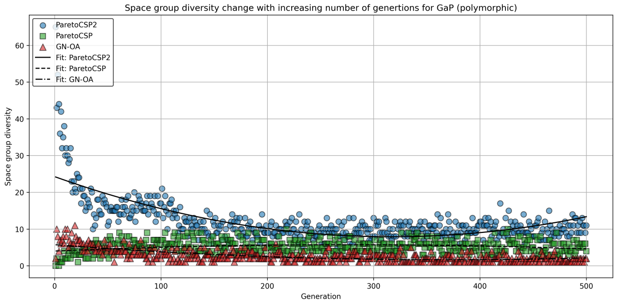

(f) GaP

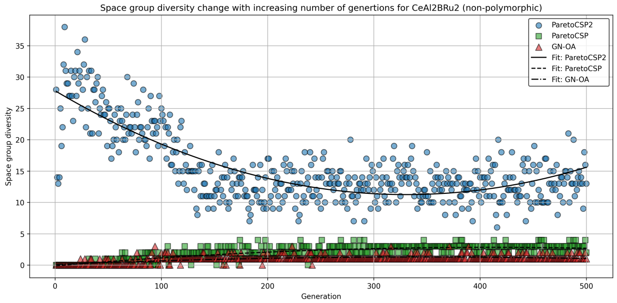

(g) $CeAl_2BRu_2$

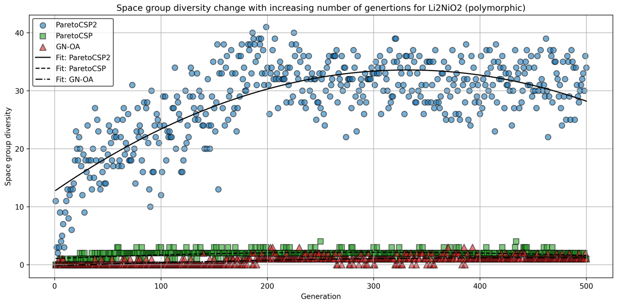

(h) Li_2NiO_2

Figure S7: Space group diversity (↑) progression of ParetoCSP2 vs ParetoCSP and GN-OA for some case studies for a population size of 100. (a), (c), (e), and (g) present sample non-polymorphic cases, and (b), (d), (f), and (h) present some polymorphic cases that show the evolution of space group diversity over 500 generations. ParetoCSP2 consistently achieves significantly higher space group diversity throughout the optimization process, while ParetoCSP and GN-OA remain limited to fewer distinct space groups. The trend lines indicate the quadratic fitted progression of space group diversity for each algorithm, highlighting ParetoCSP2's superior exploration capability and adaptability in both non-polymorphic and polymorphic cases.

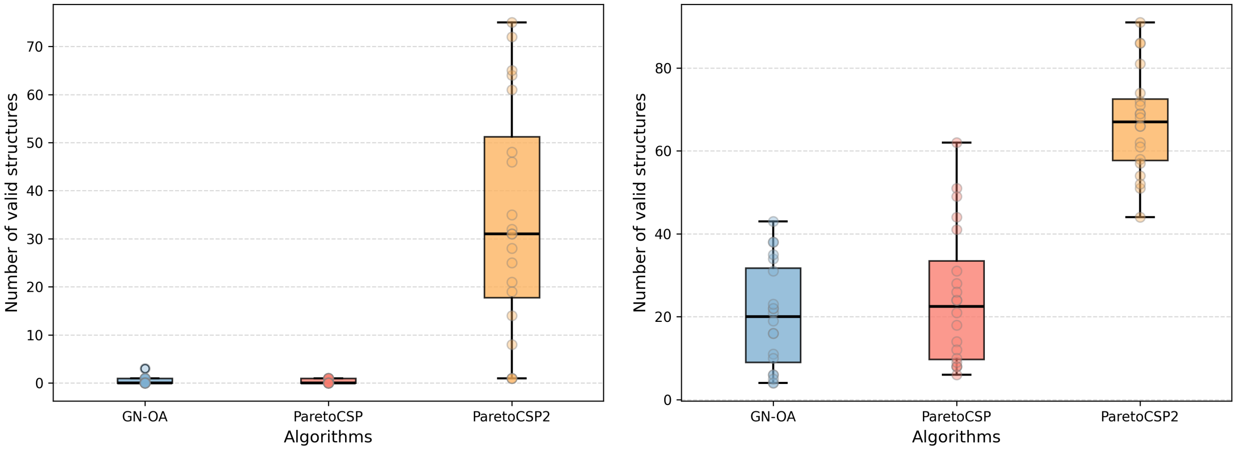

(a) **(b)**

Figure S8: Valid structure generation at early and final stages. Box plots compare the number of valid structures produced by each algorithm after the (a) first generation and (b) after 500 generations. ParetoCSP2 shows a marked improvement in valid structure count from the beginning and maintains superiority throughout, demonstrating the effectiveness of its initialization strategy and consistent relaxation. GN-OA and ParetoCSP, in contrast, struggle to generate feasible structures, limiting their ability to explore the configuration space effectively.

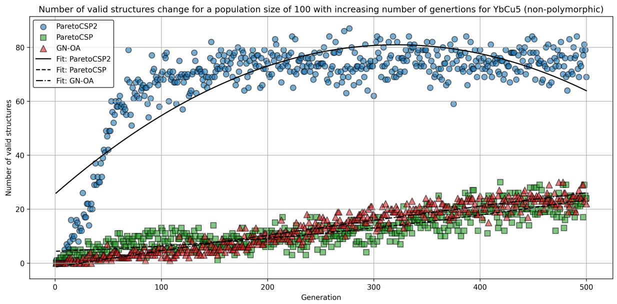

(a) $YbCu_5$

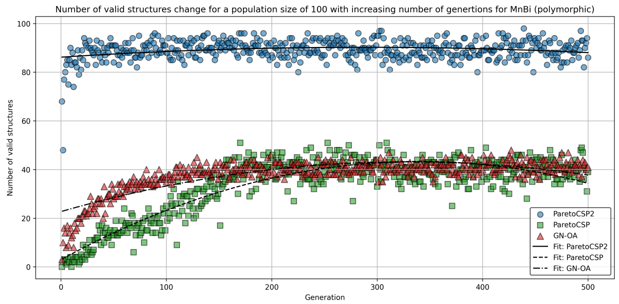

(b) MnBi

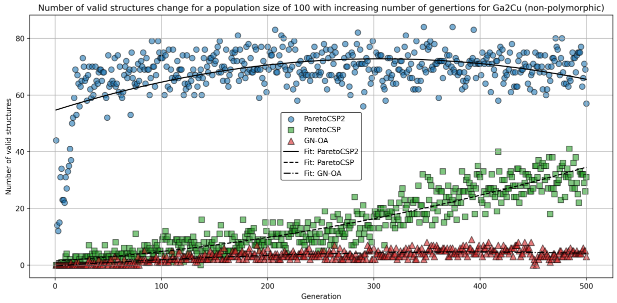

(c) Ga_2Cu

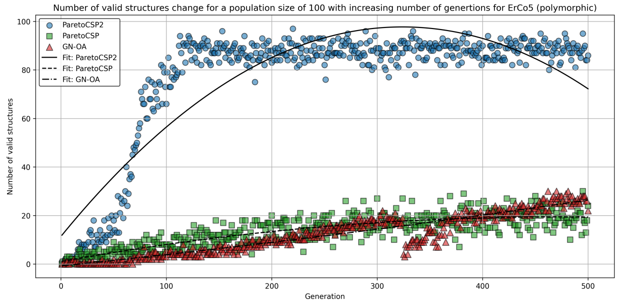

(d) $ErCo_5$

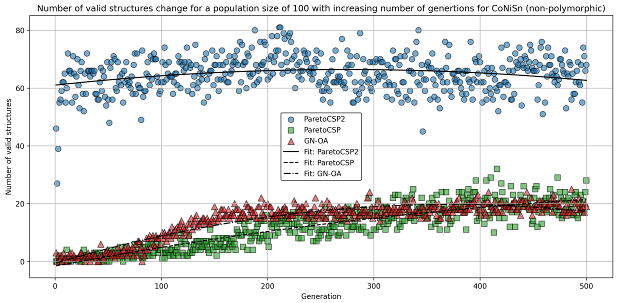

(e) CoNiSn

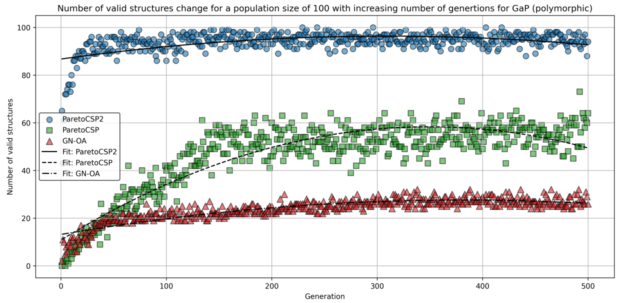

(f) GaP

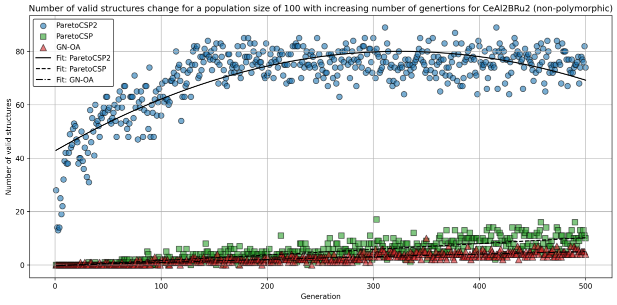

(g) $CeAl_2BRu_2$

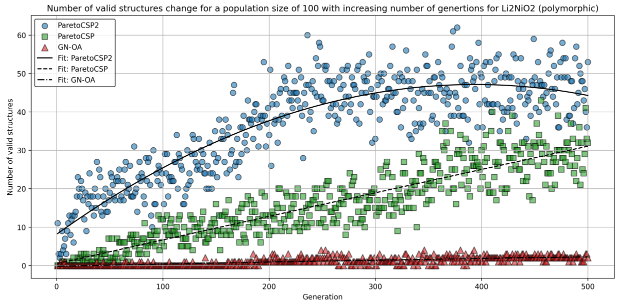

(h) Li_2NiO_2

Figure S9: Valid structure count (↑) progression of ParetoCSP2 vs ParetoCSP and GN-OA for two case studies for a population size of 100.
(a), (c), (e), and (g) present sample non-polymorphic cases, and (b), (d), (f), and (h) present some polymorphic cases that show the evolution of valid structure count over 500 generations. In both cases, all three algorithms achieved increasing number of valid structures, but the increase in ParetoCSP2 is significantly higher than that of ParetoCSP and GN-OA. The trend lines represent the quadratic fitted progression of valid structure counts, demonstrating the effectiveness of ParetoCSP2's PyXtal-based initialization and space group-specific optimization criterion in facilitating better exploration and preservation of valid structures over generations.

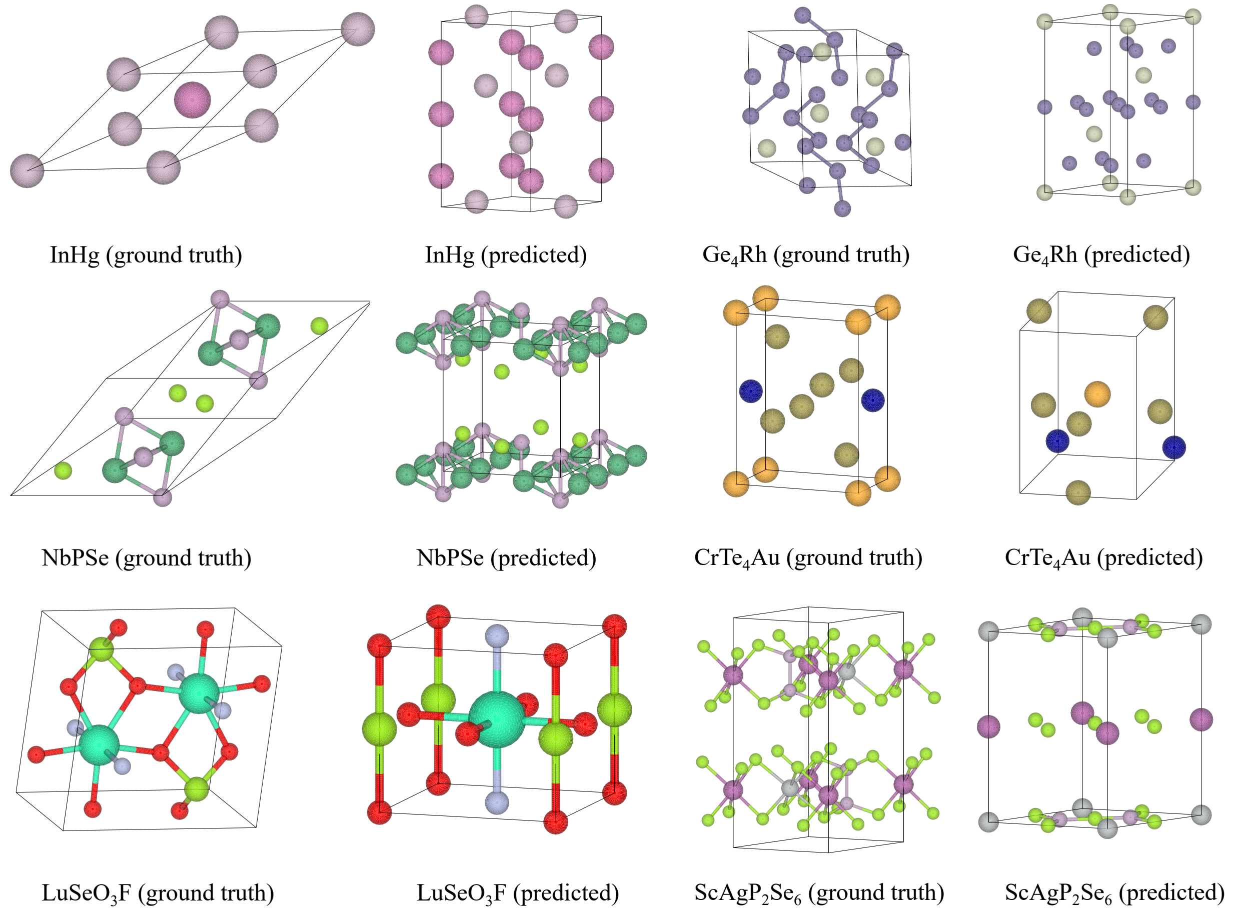

Figure S10: Sample failure predictions by ParetoCSP2. Despite ParetoCSP2's success, it struggled with predicting structures of lower-symmetry crystals, such as monoclinic and orthorhombic crystals.

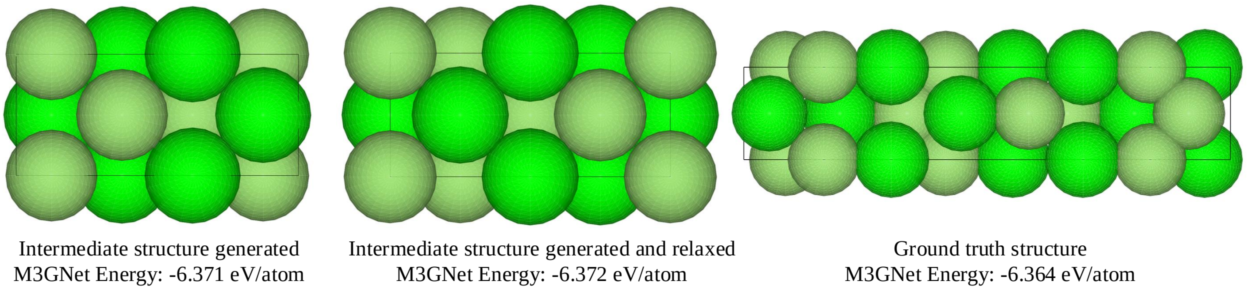

Figure S11: Example of an incorrect energy prediction by M3GNet. A case of ZrGa is shown, where it assigned an intermediate structure (left) and its relaxed form (middle) an energy value greater than that of its most stable form (right), and in the process misguided the algorithm to predict the exact structure. This underscores the necessity of designing more accurate ML IAPs to implement better CSP algorithms.

S3 Supplementary Tables

Table S1: Details of the selected polymorphic chemical formulas in this work. The formulas represent the number of atoms present in the unit cell of the polymorphic structures. The formulas are divided by a horizontal line based on the number of polymorphs they can form. We notice that a crystal can form different polymorphs with the same space group number, a measure we handled in our algorithm

Unit cell formula	Number of polymoprhs	Space groups
Ce_1Tl_1	2	123, 221
$Er_1Ag_1S_2$	2	123, 166
Er_1N_1	2	221, 225
Fe_3Sn_1	2	123, 221
$Li_1Pd_1N_1$	2	123, 123
Mg_1Ti_1	2	187, 221
Ni_1Hg_1	2	123, 221
Re_1Pt_1	2	166, 187
Tm_1Te_1	2	221, 225
V_1Fe_1	2	65, 221
Y_1As_1	2	221, 225
Si_1C_1	2	216, 225
Sr_1Te_1	2	221, 225
Y_1P_1	2	221, 225
Sn_1P_1	2	107, 225
Ce_1Bi_1	3	123, 221, 225
Mg_1O_1	3	187, 221, 225
Ta_1Ru_1	3	65, 123, 221
Al_1As_1	3	123, 216, 225
Ba_1C_2	3	123, 139, 166
Hf_1N_1	3	216, 221, 225
Mn_1Sb_1	3	216, 221, 225
Sm_1Hg_2	3	12, 164, 191
La_3Mg_1	4	25, 44, 139, 221
Mg_1Sn_1	4	123, 187, 221, 225

Ag_2O_2	4	15, 15, 15, 131
Cd_1Cl_2	4	1, 2, 115, 166
Ce_1Mg_1	4	166, 187, 221, 225
Mg_2Zn_2	4	12, 51, 59, 164
Os_1C_1	4	187, 216, 221, 225
Ti_1S_2	4	12, 12, 139, 191
Ge_1Te_1	5	8, 160, 160, 221, 225
Li_1Mg_1	5	65, 123, 166, 187, 221
Mn_1O_2	5	2, 123, 164, 166, 166
Cu_3N_1	5	71, 139, 164, 221, 221
Mg_1Sb_1	5	123, 166, 187, 221, 225
Mo_1N_1	5	187, 191, 216, 221, 225
Rb_1S_1	5	12, 25, 123, 221, 225
Mg_3Ga_1	5	25, 139, 187, 221, 225
Mg_1Ga_3	6	25, 44, 139, 187, 221, 225
Mg_1Al_1	6	123, 166, 187, 216, 221, 225
Mg_1Cd_1	6	123, 166, 187, 216, 221, 225
Mg_1Ga_1	6	123, 166, 187, 216, 221, 225
Ce_3Mg_1	6	25, 44, 139, 187, 221, 225
Y_3Mg_1	7	25, 44, 44, 139, 187, 221, 225
In_1Sb_1	7	25, 44, 119, 123, 216, 221, 225
Ca_3Mg_1	7	25, 44, 44, 139, 187, 221, 225
Nb_1S_2	8	5, 38, 42, 139, 160, 164, 166, 187
V_1S_2	8	1, 1, 1, 2, 47, 164, 166, 166
Na_1N_3	11	1, 5, 12, 12, 12, 12, 12, 160, 166, 221

Table S2: Details of the 120 benchmark crystals used in this work. The binary, the ternary, and the quaternary crystals are separated by single horizontal lines.

Material ID	Reduced formula	Unit cell formula	Crystal system	Space group number	Formation energy (eV/atom)
mp-30497	$TbCd_2$	Tb_1Cd_2	Hexagonal	191	-0.328
mp-2209	$CeGa_2$	Ce_1Ga_2	Hexagonal	191	-0.584
mp-2740	$ErCo_5$	Er_1Co_5	Hexagonal	191	-0.124
mp-1607	$YbCu_5$	Yb_1Cu_5	Hexagonal	191	-0.227
mp-1566	$SmFe_5$	Sm_1Fe_5	Hexagonal	191	0.006
mp-30725	YHg_2	Y_1Hg_2	Hexagonal	191	-0.399
mp-2510	ZrHg	Zr_2Hg_2	Tetragonal	123	-0.170
mp-2067	$ThGa_2$	Th_2Ga_4	Tetragonal	141	-0.639
mp-13449	$YbGa_2$	Yb_1Ga_2	Hexagonal	191	-0.548
mp-2451	YAl_3	Y_3Al_9	Trigonal	166	-0.434
mp-1004	Nb_3Si	Nb_3Si_1	Cubic	221	-0.234
mp-8882	GaP	Ga_2P_2	Hexagonal	186	-0.399
mp-9761	ReO_3	Re_2O_6	Hexagonal	182	-2.040
mp-2231	SnS	Sn_4S_4	Orthorhombic	62	-0.778
mp-568382	MnBi	Mn_2Bi_2	Hexagonal	194	0.809
mp-11359	Ga_2Cu	Ga_2Cu_1	Tetragonal	123	-0.076
mp-1827	$SrGa_4$	Sr_2Ga_8	Tetragonal	139	-0.419
mp-30746	YIr	Y_1Ir_1	Cubic	221	-0.777
mp-2735	PaO	Pa_1O_1	Cubic	225	-2.782
mp-1121	GaCo	Ga_1Co_1	Cubic	221	-0.282
mp-20225	$CePb_3$	Ce_1Pb_3	Cubic	221	-0.349
mp-1231	Cr_3Ga	Cr_6Ga_2	Cubic	223	-0.042
mp-2334	DyCu	Dy_1Cu_1	Cubic	221	-0.248
mp-1169	ScCu	Sc_1Cu_1	Cubic	221	-0.281
mp-20176	$DyPb_3$	Dy_1Pb_3	Cubic	221	-0.278
mp-2226	DyPd	Dy_1Pd_1	Cubic	221	-0.896
mp-788	CoTe	Co_2Te_2	Hexagonal	194	-0.065
mp-20132	InHg	In_3Hg_3	Trigonal	166	0.005

mp-13452	$BePd_2$	Be_2Pd_4	Tetragonal	139	-0.508
mp-1995	PrC_2	Pr_2C_4	Tetragonal	139	-0.103
mp-24658	SmH_2	Sm_4H_8	Cubic	225	-0.751
mp-30501	Ti_2Cd	Ti_4Cd_2	Tetragonal	139	-0.075
mp-30789	U_2Mo	U_4Mo_2	Tetragonal	139	0.069
mp-13181	LaF_3	La_4F_{12}	Cubic	225	-4.332
mp-2731	$TiGa_3$	Ti_2Ga_6	Tetragonal	139	-0.374
mp-454	$NaGa_4$	Na_2Ga_8	Tetragonal	139	-0.146
mp-1071835	$DyCu_2$	Dy_4Cu_8	Orthorhombic	74	-0.270
mp-861	Hf_2Ni	Hf_8Ni_4	Tetragonal	140	-0.340
mp-2129	$NdGe_2$	Nd_4Ge_8	Tetragonal	141	-0.608
mp-2387	Th_2Zn	Th_8Zn_4	Tetragonal	140	-0.157
mp-11251	Mg_3Au	Mg_6Au_2	Hexagonal	194	-0.202
mp-30682	ZrGa	Zr_8Ga_8	Tetragonal	141	-0.626
mp-11449	$HfMn_2$	Hf_4Mn_8	Hexagonal	194	-0.255
mp-640079	Mn_3Au	Mn_9Au_3	Tetragonal	123	0.301
mp-1102936	Ta_2Fe	Ta_8Fe_4	Hexagonal	193	0.363
mp-1104286	Ge_4Rh	$Ge_{12}Rh_3$	Trigonal	152	-0.200
mp-1106395	Pr_3Ir	$Pr_{12}Ir_4$	Orthorhombic	62	-0.426
mp-1105958	Yb_3Co	$Yb_{12}Co_4$	Orthorhombic	62	0.051
mp-19183	Li_2NiO_2	$Li_2Ni_1O_2$	Orthorhombic	71	-1.552
mp-19227	$YMnO_3$	$Y_2Mn_2O_6$	Hexagonal	194	-3.022
mp-29241	Ca_3SnO	$Ca_3Sn_1O_1$	Cubic	221	-1.773
mp-20237	CoNiSn	$Co_2Ni_2Sn_2$	Hexagonal	194	-0.014
mp-11396	$NdGa_2Ni$	$Nd_2Ga_4Ni_2$	Orthorhombic	65	-0.623
mp-9570	$CaCd_2P_2$	$Ca_1Cd_2P_2$	Trigonal	164	-0.571
mp-30493	$ZnCdPt_2$	$Zn_1Cd_1Pt_2$	Tetragonal	123	-0.448
mp-1106349	$SmPd_3S_4$	$Sm_2Pd_6S_8$	Cubic	223	-1.239
mp-27450	$KCuCl_3$	$K_4Cu_4Cl_{12}$	Monoclinic	14	-1.451
mp-11390	LiGaSi	$Li_4Ga_4Si_4$	Cubic	216	-0.164
mp-4552	$ErInCu_2$	$Er_4In_4Cu_8$	Cubic	225	-0.323
mp-20730	$HfCo_2Sn$	$Hf_4Co_8Sn_4$	Cubic	225	-0.360

mp-21211	$InFeCo_2$	$In_4Fe_4Co_8$	Cubic	225	0.064
mp-4326	$KErS_2$	$K_3Er_3S_6$	Trigonal	166	-2.213
mp-30591	Li_2CuSn	$Li_8Cu_4Sn_4$	Cubic	216	-0.334
mp-30648	$LiMg_2Ga$	$Li_4Mg_8Ga_4$	Cubic	225	-0.128
mp-4972	$LuInCu_2$	$Lu_4In_4Cu_8$	Cubic	225	-0.315
mp-5181	$LuSnPd_2$	$Lu_4Sn_4Pd_8$	Cubic	225	-0.906
mp-20389	Na_2CdPb	$Na_8Cd_4Pb_4$	Cubic	216	-0.154
mp-30580	$SrGaCu_2$	$Sr_3Ga_3Cu_6$	Trigonal	166	-0.258
mp-30555	$TaGaCo_2$	$Ta_4Ga_4Co_8$	Cubic	225	-0.360
mp-24012	HoHSe	$Ho_1H_1Se_1$	Hexagonal	187	-1.481
mp-5452	$CeCu_2Si_2$	$Ce_2Cu_4Si_4$	Tetragonal	139	-0.576
mp-3147	$ErSi_2Au_2$	$Er_2Si_4Au_4$	Tetragonal	139	-0.520
mp-13405	$LuMn_2Ge_2$	$Lu_2Mn_4Ge_4$	Tetragonal	139	-0.412
mp-30805	$SrNiSn_3$	$Sr_2Ni_2Sn_6$	Tetragonal	107	-0.446
mp-12743	$CrTe_4Au$	$Cr_1Te_4Au_1$	Monoclinic	10	-0.146
mp-7524	NbPSe	$Nb_4P_4Se_4$	Orthorhombic	71	-0.698
mp-11435	$ErTi_2Ga_4$	$Er_2Ti_4Ga_8$	Tetragonal	139	-0.532
mp-7095	$CeCoSi_2$	$Ce_4Co_4Si_8$	Orthorhombic	63	-0.789
mp-5615	CaAgAs	$Ca_3Ag_3As_3$	Hexagonal	189	-0.791
mp-30733	HoSnPt	$Ho_3Sn_3Pt_3$	Hexagonal	189	-1.095
mp-16747	LuAgPb	$Lu_3Ag_3Pb_3$	Hexagonal	189	-0.299
mp-13171	YMgCu	$Y_3Mg_3Cu_3$	Hexagonal	189	-0.216
mp-7550	$CeNbO_4$	$Ce_2Nb_2O_8$	Monoclinic	15	-3.484
mp-12931	$SmTaO_4$	$Sm_2Ta_2O_8$	Monoclinic	13	-3.712
mp-7913	$BeSiN_2$	$Be_4Si_4N_8$	Orthorhombic	33	-1.413
mp-1106406	Ce_2SnS_5	$Ce_4Sn_2S_{10}$	Orthorhombic	55	-1.837
mp-1106117	La_5AgPb_3	$La_{10}Ag_2Pb_6$	Hexagonal	193	-0.543
mp-1106245	Zr_5AlSb_3	$Zr_{10}Al_2Sb_6$	Hexagonal	193	-0.712
mp-557997	$CaSeO_3$	$Ca_4Se_4O_{12}$	Monoclinic	14	-2.317
mp-19140	K_3MnO_4	$K_6Mn_2O_8$	Tetragonal	121	-1.783
mp-6258	$CeCr_2Si_2C$	$Ce_1Cr_2Si_2C_1$	Tetragonal	123	-0.492
mp-726253	$RbLi_3S_2O_9$	$Rb_1Li_3S_2O_9$	Triclinic	1	-1.65

mp-23126	KAs_4IO_6	$K_1As_4I_1O_6$	Hexagonal	191	-1.643
mp-12515	LaZnSbO	$La_2Zn_2Sb_2O_2$	Tetragonal	129	-1.937
mp-1071272	$CeAl_2BRu_2$	$Ce_1Al_2B_1Ru_2$	Tetragonal	123	-0.614
mp-1213726	CrFeCoSi	$Cr_1Fe_1Co_1Si_1$	Cubic	216	-0.293
mp-552674	ZrTaNO	$Zr_1Ta_1N_1O_1$	Hexagonal	187	-1.394
mp-7554	LiMgSnAu	$Li_4Mg_4Sn_4Au_4$	Cubic	216	-0.384
mp-11806	LiMgSnPt	$Li_4Mg_4Sn_4Pt_4$	Cubic	216	-0.671
mp-6794	LaB_2Rh_2C	$La_2B_4Rh_4C_2$	Tetragonal	139	-0.598
mp-6140	$PrNi_2B_2C$	$Pr_2Ni_4B_4C_2$	Tetragonal	139	-0.433
mp-545788	Ba_3ZnN_2O	$Ba_3Zn_1N_2O_1$	Tetragonal	123	-1.214
mp-13287	BaCuTeF	$Ba_2Cu_2Te_2F_2$	Tetragonal	129	-2.033
mp-1221640	MnAlCuPd	$Mn_1Al_1Cu_1Pd_1$	Tetragonal	99	-0.301
mp-546790	LaCuTeO	$La_2Cu_2Te_2O_2$	Tetragonal	129	-2.076
mp-18745	LaMnSbO	$La_2Mn_2Sb_2O_2$	Tetragonal	129	-1.840
mp-20349	SmFeAsO	$Sm_2Fe_2As_2O_2$	Tetragonal	129	-1.772
mp-12444	SrCuSF	$Sr_2Cu_2S_2F_2$	Tetragonal	129	-2.351
mp-20807	SrFeAsF	$Sr_2Fe_2As_2F_2$	Tetragonal	129	-1.809
mp-19118	$BaNd_2CoO_5$	$Ba_2Nd_4Co_2O_{10}$	Orthorhombic	71	-3.012
mp-23127	Ba_2BiSbO_6	$Ba_4Bi_2Sb_2O_{12}$	Monoclinic	12	-2.463
mp-21348	Ba_2EuTaO_6	$Ba_4Eu_2Ta_2O_{12}$	Tetragonal	87	-3.346
mp-19274	$BaPrMn_2O_6$	$Ba_1Pr_1Mn_2O_6$	Tetragonal	123	-2.693
mp-1095437	$LuSeO_3F$	$Lu_2Se_2O_6F_2$	Monoclinic	11	-2.875
mp-1106310	Cu_2SiHgS_4	$Cu_4Si_2Hg_2S_8$	Orthorhombic	31	-0.715
mp-1105386	YbH_3CN_3	$Yb_2H_6C_2N_6$	Hexagonal	176	-0.795
mp-651268	$Fe_2Cu_6SnS_8$	$Fe_2Cu_6Sn_1S_8$	Tetragonal	115	-0.591
mp-1105110	$Sr_2Bi_2Se_3O_2$	$Sr_4Bi_4Se_6O_4$	Monoclinic	14	-1.83
mp-1105674	$Mg_2BeB_2Ir_5$	$Mg_4Be_2B_4Ir_{10}$	Tetragonal	127	-0.521
mp-13383	$ScAgP_2Se_6$	$Sc_2Ag_2P_4Se_{12}$	Trigonal	163	-0.524

Table S3: Successful and unsuccessful space group matches of all three algorithms for each benchmark crystal. The binary, the ternary, and the quaternary crystals are separated by single horizontal lines.

Material ID	Reduced formula	Unit cell formula	ParetoCSP2	ParetoCSP	GN-OA
mp-30497	$TbCd_2$	Tb_1Cd_2	✓	✓	✓
mp-2209	$CeGa_2$	Ce_1Ga_2	✓	✓	✓
mp-2740	$ErCo_5$	Er_1Co_5	✓	✓	✓
mp-1607	$YbCu_5$	Yb_1Cu_5	✓	✓	✗
mp-1566	$SmFe_5$	Sm_1Fe_5	✓	✗	✓
mp-30725	YHg_2	Y_1Hg_2	✓	✗	✗
mp-2510	ZrHg	Zr_2Hg_2	✓	✗	✗
mp-2067	$ThGa_2$	Th_2Ga_4	✓	✗	✗
mp-13449	$YbGa_2$	Yb_1Ga_2	✓	✗	✗
mp-2451	YAl_3	Y_3Al_9	✓	✓	✗
mp-1004	Nb_3Si	Nb_3Si_1	✓	✗	✗
mp-8882	GaP	Ga_2P_2	✓	✓	✓
mp-9761	ReO_3	Re_2O_6	✓	✓	✗
mp-2231	SnS	Sn_4S_4	✓	✓	✗
mp-568382	MnBi	Mn_2Bi_2	✓	✓	✗
mp-11359	Ga_2Cu	Ga_2Cu_1	✓	✓	✗
mp-1827	$SrGa_4$	Sr_2Ga_8	✓	✗	✗
mp-30746	YIr	Y_1Ir_1	✓	✓	✗
mp-2735	PaO	Pa_1O_1	✓	✗	✗
mp-1121	GaCo	Ga_1Co_1	✓	✗	✗
mp-20225	$CePb_3$	Ce_1Pb_3	✓	✓	✗
mp-1231	Cr_3Ga	Cr_6Ga_2	✓	✓	✓
mp-2334	DyCu	Dy_1Cu_1	✓	✓	✗
mp-1169	ScCu	Sc_1Cu_1	✓	✓	✗
mp-20176	$DyPb_3$	Dy_1Pb_3	✓	✓	✗
mp-2226	DyPd	Dy_1Pd_1	✓	✓	✗
mp-788	CoTe	Co_2Te_2	✓	✗	✓

mp-20132	InHg	In_3Hg_3	✗	✗	✗
mp-13452	$BePd_2$	Be_2Pd_4	✗	✗	✗
mp-1995	PrC_2	Pr_2C_4	✗	✗	✗
mp-24658	SmH_2	Sm_4H_8	✓	✗	✗
mp-30501	Ti_2Cd	Ti_4Cd_2	✗	✗	✗
mp-30789	U_2Mo	U_4Mo_2	✗	✗	✗
mp-13181	LaF_3	La_4F_{12}	✓	✗	✗
mp-2731	$TiGa_3$	Ti_2Ga_6	✓	✗	✗
mp-454	$NaGa_4$	Na_2Ga_8	✓	✗	✗
mp-1071835	$DyCu_2$	Dy_4Cu_8	✗	✗	✗
mp-861	Hf_2Ni	Hf_8Ni_4	✗	✗	✗
mp-2129	$NdGe_2$	Nd_4Ge_8	✓	✗	✗
mp-2387	Th_2Zn	Th_8Zn_4	✗	✗	✗
mp-11251	Mg_3Au	Mg_6Au_2	✗	✗	✗
mp-30682	ZrGa	Zr_8Ga_8	✗	✗	✗
mp-11449	$HfMn_2$	Hf_4Mn_8	✗	✗	✗
mp-640079	Mn_3Au	Mn_9Au_3	✗	✗	✗
mp-1102936	Ta_2Fe	Ta_8Fe_4	✗	✗	✗
mp-1104286	Ge_4Rh	$Ge_{12}Rh_3$	✗	✗	✗
mp-1106395	Pr_3Ir	$Pr_{12}Ir_4$	✗	✗	✗
mp-1105958	Yb_3Co	$Yb_{12}Co_4$	✗	✗	✗
mp-19183	Li_2NiO_2	$Li_2Ni_1O_2$	✓	✗	✗
mp-19227	$YMnO_3$	$Y_2Mn_2O_6$	✓	✗	✗
mp-29241	Ca_3SnO	$Ca_3Sn_1O_1$	✓	✓	✗
mp-20237	CoNiSn	$Co_2Ni_2Sn_2$	✓	✓	✗
mp-11396	$NdGa_2Ni$	$Nd_2Ga_4Ni_2$	✓	✗	✗
mp-9570	$CaCd_2P_2$	$Ca_1Cd_2P_2$	✓	✗	✗
mp-30493	$ZnCdPt_2$	$Zn_1Cd_1Pt_2$	✓	✓	✗
mp-1106349	$SmPd_3S_4$	$Sm_2Pd_6S_8$	✓	✓	✓
mp-27450	$KCuCl_3$	$K_4Cu_4Cl_{12}$	✗	✗	✗
mp-11390	LiGaSi	$Li_4Ga_4Si_4$	✓	✗	✗
mp-4552	$ErInCu_2$	$Er_4In_4Cu_8$	✓	✗	✗

mp-20730	$HfCo_2Sn$	$Hf_4Co_8Sn_4$	✓	✗	✗
mp-21211	$InFeCo_2$	$In_4Fe_4Co_8$	✓	✗	✗
mp-4326	$KErS_2$	$K_3Er_3S_6$	✓	✗	✗
mp-30591	Li_2CuSn	$Li_8Cu_4Sn_4$	✓	✗	✗
mp-30648	$LiMg_2Ga$	$Li_4Mg_8Ga_4$	✓	✗	✗
mp-4972	$LuInCu_2$	$Lu_4In_4Cu_8$	✓	✗	✗
mp-5181	$LuSnPd_2$	$Lu_4Sn_4Pd_8$	✓	✗	✗
mp-20389	Na_2CdPb	$Na_8Cd_4Pb_4$	✓	✗	✗
mp-30580	$SrGaCu_2$	$Sr_3Ga_3Cu_6$	✗	✗	✗
mp-30555	$TaGaCo_2$	$Ta_4Ga_4Co_8$	✓	✗	✗
mp-24012	HoHSe	$Ho_1H_1Se_1$	✓	✗	✗
mp-5452	$CeCu_2Si_2$	$Ce_2Cu_4Si_4$	✗	✗	✗
mp-3147	$ErSi_2Au_2$	$Er_2Si_4Au_4$	✗	✗	✗
mp-13405	$LuMn_2Ge_2$	$Lu_2Mn_4Ge_4$	✗	✗	✗
mp-30805	$SrNiSn_3$	$Sr_2Ni_2Sn_6$	✗	✗	✗
mp-12743	$CrTe_4Au$	$Cr_1Te_4Au_1$	✗	✗	✗
mp-7524	NbPSe	$Nb_4P_4Se_4$	✗	✗	✗
mp-11435	$ErTi_2Ga_4$	$Er_2Ti_4Ga_8$	✓	✗	✗
mp-7095	$CeCoSi_2$	$Ce_4Co_4Si_8$	✗	✗	✗
mp-5615	CaAgAs	$Ca_3Ag_3As_3$	✗	✗	✗
mp-30733	HoSnPt	$Ho_3Sn_3Pt_3$	✗	✗	✗
mp-16747	LuAgPb	$Lu_3Ag_3Pb_3$	✗	✗	✗
mp-13171	YMgCu	$Y_3Mg_3Cu_3$	✗	✗	✗
mp-7550	$CeNbO_4$	$Ce_2Nb_2O_8$	✗	✗	✗
mp-12931	$SmTaO_4$	$Sm_2Ta_2O_8$	✗	✗	✗
mp-7913	$BeSiN_2$	$Be_4Si_4N_8$	✓	✗	✗
mp-1106406	Ce_2SnS_5	$Ce_4Sn_2S_{10}$	✗	✗	✗
mp-1106117	La_5AgPb_3	$La_{10}Ag_2Pb_6$	✗	✗	✗
mp-1106245	Zr_5AlSb_3	$Zr_{10}Al_2Sb_6$	✗	✗	✗
mp-557997	$CaSeO_3$	$Ca_4Se_4O_{12}$	✗	✗	✗
mp-19140	K_3MnO_4	$K_6Mn_2O_8$	✗	✗	✗
mp-6258	$CeCr_2Si_2C$	$Ce_1Cr_2Si_2C_1$	✓	✗	✗

mp-726253	$RbLi_3S_2O_9$	$Rb_1Li_3S_2O_9$	✓	✓	✗
mp-23126	KAs_4IO_6	$K_1As_4I_1O_6$	✓	✗	✗
mp-12515	LaZnSbO	$La_2Zn_2Sb_2O_2$	✓	✗	✗
mp-1071272	$CeAl_2BRu_2$	$Ce_1Al_2B_1Ru_2$	✓	✗	✗
mp-1213726	CrFeCoSi	$Cr_1Fe_1Co_1Si_1$	✓	✓	✗
mp-552674	ZrTaNO	$Zr_1Ta_1N_1O_1$	✓	✓	✗
mp-7554	LiMgSnAu	$Li_4Mg_4Sn_4Au_4$	✓	✗	✗
mp-11806	LiMgSnPt	$Li_4Mg_4Sn_4Pt_4$	✓	✗	✗
mp-6794	LaB_2Rh_2C	$La_2B_4Rh_4C_2$	✓	✗	✗
mp-6140	$PrNi_2B_2C$	$Pr_2Ni_4B_4C_2$	✓	✗	✗
mp-545788	Ba_3ZnN_2O	$Ba_3Zn_1N_2O_1$	✗	✗	✗
mp-13287	BaCuTeF	$Ba_2Cu_2Te_2F_2$	✗	✗	✗
mp-1221640	MnAlCuPd	$Mn_1Al_1Cu_1Pd_1$	✓	✗	✗
mp-546790	LaCuTeO	$La_2Cu_2Te_2O_2$	✓	✗	✗
mp-18745	LaMnSbO	$La_2Mn_2Sb_2O_2$	✗	✗	✗
mp-20349	SmFeAsO	$Sm_2Fe_2As_2O_2$	✗	✗	✗
mp-12444	SrCuSF	$Sr_2Cu_2S_2F_2$	✗	✗	✗
mp-20807	SrFeAsF	$Sr_2Fe_2As_2F_2$	✗	✗	✗
mp-19118	$BaNd_2CoO_5$	$Ba_2Nd_4Co_2O_{10}$	✗	✗	✗
mp-23127	Ba_2BiSbO_6	$Ba_4Bi_2Sb_2O_{12}$	✗	✗	✗
mp-21348	Ba_2EuTaO_6	$Ba_4Eu_2Ta_2O_{12}$	✗	✗	✗
mp-19274	$BaPrMn_2O_6$	$Ba_1Pr_1Mn_2O_6$	✗	✗	✗
mp-1095437	$LuSeO_3F$	$Lu_2Se_2O_6F_2$	✗	✗	✗
mp-1106310	Cu_2SiHgS_4	$Cu_4Si_2Hg_2S_8$	✗	✗	✗
mp-1105386	YbH_3CN_3	$Yb_2H_6C_2N_6$	✗	✗	✗
mp-651268	$Fe_2Cu_6SnS_8$	$Fe_2Cu_6Sn_1S_8$	✗	✗	✗
mp-1105110	$Sr_2Bi_2Se_3O_2$	$Sr_4Bi_4Se_6O_4$	✗	✗	✗
mp-1105674	$Mg_2BeB_2Ir_5$	$Mg_4Be_2B_4Ir_{10}$	✗	✗	✗
mp-13383	$ScAgP_2Se_6$	$Sc_2Ag_2P_4Se_{12}$	✗	✗	✗

Table S4: Successful and unsuccessful StructureMatcher matches of all three algorithms for each benchmark crystal. The binary, the ternary, and the quaternary crystals are separated by single horizontal lines.

Material ID	Reduced formula	Unit cell formula	ParetoCSP2	ParetoCSP	GN-OA
mp-30497	$TbCd_2$	Tb_1Cd_2	✓	✓	✓
mp-2209	$CeGa_2$	Ce_1Ga_2	✓	✓	✓
mp-2740	$ErCo_5$	Er_1Co_5	✓	✓	✓
mp-1607	$YbCu_5$	Yb_1Cu_5	✓	✓	✗
mp-1566	$SmFe_5$	Sm_1Fe_5	✓	✗	✓
mp-30725	YHg_2	Y_1Hg_2	✓	✗	✓
mp-2510	ZrHg	Zr_2Hg_2	✓	✗	✗
mp-2067	$ThGa_2$	Th_2Ga_4	✓	✗	✗
mp-13449	$YbGa_2$	Yb_1Ga_2	✓	✗	✗
mp-2451	YAl_3	Y_3Al_9	✓	✗	✗
mp-1004	Nb_3Si	Nb_3Si_1	✓	✓	✗
mp-8882	GaP	Ga_2P_2	✓	✓	✓
mp-9761	ReO_3	Re_2O_6	✓	✓	✗
mp-2231	SnS	Sn_4S_4	✓	✓	✗
mp-568382	MnBi	Mn_2Bi_2	✓	✓	✗
mp-11359	Ga_2Cu	Ga_2Cu_1	✓	✓	✗
mp-1827	$SrGa_4$	Sr_2Ga_8	✗	✗	✗
mp-30746	YIr	Y_1Ir_1	✓	✓	✗
mp-2735	PaO	Pa_1O_1	✓	✗	✗
mp-1121	GaCo	Ga_1Co_1	✓	✗	✗
mp-20225	$CePb_3$	Ce_1Pb_3	✓	✓	✗
mp-1231	Cr_3Ga	Cr_6Ga_2	✓	✓	✓
mp-2334	DyCu	Dy_1Cu_1	✓	✓	✗
mp-1169	ScCu	Sc_1Cu_1	✓	✓	✗
mp-20176	$DyPb_3$	Dy_1Pb_3	✓	✓	✗
mp-2226	DyPd	Dy_1Pd_1	✓	✓	✗
mp-788	CoTe	Co_2Te_2	✓	✗	✓

mp-20132	InHg	In_3Hg_3	✗	✗	✗
mp-13452	$BePd_2$	Be_2Pd_4	✗	✗	✗
mp-1995	PrC_2	Pr_2C_4	✗	✗	✗
mp-24658	SmH_2	Sm_4H_8	✓	✗	✗
mp-30501	Ti_2Cd	Ti_4Cd_2	✗	✗	✗
mp-30789	U_2Mo	U_4Mo_2	✗	✗	✗
mp-13181	LaF_3	La_4F_{12}	✓	✗	✗
mp-2731	$TiGa_3$	Ti_2Ga_6	✓	✗	✗
mp-454	$NaGa_4$	Na_2Ga_8	✗	✗	✗
mp-1071835	$DyCu_2$	Dy_4Cu_8	✗	✗	✗
mp-861	Hf_2Ni	Hf_8Ni_4	✗	✗	✗
mp-2129	$NdGe_2$	Nd_4Ge_8	✗	✗	✗
mp-2387	Th_2Zn	Th_8Zn_4	✗	✗	✗
mp-11251	Mg_3Au	Mg_6Au_2	✗	✗	✗
mp-30682	ZrGa	Zr_8Ga_8	✗	✗	✗
mp-11449	$HfMn_2$	Hf_4Mn_8	✗	✗	✗
mp-640079	Mn_3Au	Mn_9Au_3	✗	✗	✗
mp-1102936	Ta_2Fe	Ta_8Fe_4	✗	✗	✗
mp-1104286	Ge_4Rh	$Ge_{12}Rh_3$	✗	✗	✗
mp-1106395	Pr_3Ir	$Pr_{12}Ir_4$	✗	✗	✗
mp-1105958	Yb_3Co	$Yb_{12}Co_4$	✗	✗	✗
mp-19183	Li_2NiO_2	$Li_2Ni_1O_2$	✓	✗	✗
mp-19227	$YMnO_3$	$Y_2Mn_2O_6$	✓	✗	✗
mp-29241	Ca_3SnO	$Ca_3Sn_1O_1$	✓	✓	✗
mp-20237	CoNiSn	$Co_2Ni_2Sn_2$	✓	✓	✗
mp-11396	$NdGa_2Ni$	$Nd_2Ga_4Ni_2$	✗	✗	✗
mp-9570	$CaCd_2P_2$	$Ca_1Cd_2P_2$	✓	✗	✗
mp-30493	$ZnCdPt_2$	$Zn_1Cd_1Pt_2$	✓	✓	✗
mp-1106349	$SmPd_3S_4$	$Sm_2Pd_6S_8$	✓	✓	✓
mp-27450	$KCuCl_3$	$K_4Cu_4Cl_{12}$	✓	✗	✗
mp-11390	LiGaSi	$Li_4Ga_4Si_4$	✓	✗	✗
mp-4552	$ErInCu_2$	$Er_4In_4Cu_8$	✓	✗	✗

mp-20730	$HfCo_2Sn$	$Hf_4Co_8Sn_4$	✓	✗	✗
mp-21211	$InFeCo_2$	$In_4Fe_4Co_8$	✓	✗	✗
mp-4326	$KErS_2$	$K_3Er_3S_6$	✓	✗	✗
mp-30591	Li_2CuSn	$Li_8Cu_4Sn_4$	✓	✗	✗
mp-30648	$LiMg_2Ga$	$Li_4Mg_8Ga_4$	✓	✗	✗
mp-4972	$LuInCu_2$	$Lu_4In_4Cu_8$	✓	✗	✗
mp-5181	$LuSnPd_2$	$Lu_4Sn_4Pd_8$	✓	✗	✗
mp-20389	Na_2CdPb	$Na_8Cd_4Pb_4$	✓	✗	✗
mp-30580	$SrGaCu_2$	$Sr_3Ga_3Cu_6$	✗	✗	✗
mp-30555	$TaGaCo_2$	$Ta_4Ga_4Co_8$	✓	✗	✗
mp-24012	HoHSe	$Ho_1H_1Se_1$	✓	✗	✗
mp-5452	$CeCu_2Si_2$	$Ce_2Cu_4Si_4$	✗	✗	✗
mp-3147	$ErSi_2Au_2$	$Er_2Si_4Au_4$	✗	✗	✗
mp-13405	$LuMn_2Ge_2$	$Lu_2Mn_4Ge_4$	✗	✗	✗
mp-30805	$SrNiSn_3$	$Sr_2Ni_2Sn_6$	✗	✗	✗
mp-12743	$CrTe_4Au$	$Cr_1Te_4Au_1$	✗	✗	✗
mp-7524	NbPSe	$Nb_4P_4Se_4$	✗	✗	✗
mp-11435	$ErTi_2Ga_4$	$Er_2Ti_4Ga_8$	✗	✗	✗
mp-7095	$CeCoSi_2$	$Ce_4Co_4Si_8$	✗	✗	✗
mp-5615	CaAgAs	$Ca_3Ag_3As_3$	✗	✗	✗
mp-30733	HoSnPt	$Ho_3Sn_3Pt_3$	✗	✗	✗
mp-16747	LuAgPb	$Lu_3Ag_3Pb_3$	✗	✗	✗
mp-13171	YMgCu	$Y_3Mg_3Cu_3$	✗	✗	✗
mp-7550	$CeNbO_4$	$Ce_2Nb_2O_8$	✗	✗	✗
mp-12931	$SmTaO_4$	$Sm_2Ta_2O_8$	✓	✗	✗
mp-7913	$BeSiN_2$	$Be_4Si_4N_8$	✓	✗	✗
mp-1106406	Ce_2SnS_5	$Ce_4Sn_2S_{10}$	✗	✗	✗
mp-1106117	La_5AgPb_3	$La_{10}Ag_2Pb_6$	✗	✗	✗
mp-1106245	Zr_5AlSb_3	$Zr_{10}Al_2Sb_6$	✗	✗	✗
mp-557997	$CaSeO_3$	$Ca_4Se_4O_{12}$	✗	✗	✗
mp-19140	K_3MnO_4	$K_6Mn_2O_8$	✗	✗	✗
mp-6258	$CeCr_2Si_2C$	$Ce_1Cr_2Si_2C_1$	✓	✓	✗

mp-726253	$RbLi_3S_2O_9$	$Rb_1Li_3S_2O_9$	✗	✗	✗
mp-23126	KAs_4IO_6	$K_1As_4I_1O_6$	✗	✗	✗
mp-12515	LaZnSbO	$La_2Zn_2Sb_2O_2$	✓	✗	✗
mp-1071272	$CeAl_2BRu_2$	$Ce_1Al_2B_1Ru_2$	✓	✗	✗
mp-1213726	CrFeCoSi	$Cr_1Fe_1Co_1Si_1$	✓	✓	✗
mp-552674	ZrTaNO	$Zr_1Ta_1N_1O_1$	✓	✓	✗
mp-7554	LiMgSnAu	$Li_4Mg_4Sn_4Au_4$	✓	✗	✗
mp-11806	LiMgSnPt	$Li_4Mg_4Sn_4Pt_4$	✓	✗	✗
mp-6794	LaB_2Rh_2C	$La_2B_4Rh_4C_2$	✓	✗	✗
mp-6140	$PrNi_2B_2C$	$Pr_2Ni_4B_4C_2$	✗	✗	✗
mp-545788	Ba_3ZnN_2O	$Ba_3Zn_1N_2O_1$	✗	✗	✗
mp-13287	BaCuTeF	$Ba_2Cu_2Te_2F_2$	✗	✗	✗
mp-1221640	MnAlCuPd	$Mn_1Al_1Cu_1Pd_1$	✓	✗	✗
mp-546790	LaCuTeO	$La_2Cu_2Te_2O_2$	✓	✗	✗
mp-18745	LaMnSbO	$La_2Mn_2Sb_2O_2$	✗	✗	✗
mp-20349	SmFeAsO	$Sm_2Fe_2As_2O_2$	✗	✗	✗
mp-12444	SrCuSF	$Sr_2Cu_2S_2F_2$	✗	✗	✗
mp-20807	SrFeAsF	$Sr_2Fe_2As_2F_2$	✗	✗	✗
mp-19118	$BaNd_2CoO_5$	$Ba_2Nd_4Co_2O_{10}$	✗	✗	✗
mp-23127	Ba_2BiSbO_6	$Ba_4Bi_2Sb_2O_{12}$	✗	✗	✗
mp-21348	Ba_2EuTaO_6	$Ba_4Eu_2Ta_2O_{12}$	✗	✗	✗
mp-19274	$BaPrMn_2O_6$	$Ba_1Pr_1Mn_2O_6$	✓	✗	✗
mp-1095437	$LuSeO_3F$	$Lu_2Se_2O_6F_2$	✗	✗	✗
mp-1106310	Cu_2SiHgS_4	$Cu_4Si_2Hg_2S_8$	✗	✗	✗
mp-1105386	YbH_3CN_3	$Yb_2H_6C_2N_6$	✗	✗	✗
mp-651268	$Fe_2Cu_6SnS_8$	$Fe_2Cu_6Sn_1S_8$	✗	✗	✗
mp-1105110	$Sr_2Bi_2Se_3O_2$	$Sr_4Bi_4Se_6O_4$	✗	✗	✗
mp-1105674	$Mg_2BeB_2Ir_5$	$Mg_4Be_2B_4Ir_{10}$	✗	✗	✗
mp-13383	$ScAgP_2Se_6$	$Sc_2Ag_2P_4Se_{12}$	✗	✗	✗

Table S5: Successful and unsuccessful space group and StructureMatcher matches (consensus) of all three algorithms for each benchmark crystal. The binary, the ternary, and the quaternary crystals are separated by single horizontal lines.

Material ID	Reduced formula	Unit cell formula	ParetoCSP2	ParetoCSP	GN-OA
mp-30497	$TbCd_2$	Tb_1Cd_2	✓	✓	✓
mp-2209	$CeGa_2$	Ce_1Ga_2	✓	✓	✓
mp-2740	$ErCo_5$	Er_1Co_5	✓	✓	✓
mp-1607	$YbCu_5$	Yb_1Cu_5	✓	✓	✗
mp-1566	$SmFe_5$	Sm_1Fe_5	✓	✗	✓
mp-30725	YHg_2	Y_1Hg_2	✓	✗	✗
mp-2510	ZrHg	Zr_2Hg_2	✓	✗	✗
mp-2067	$ThGa_2$	Th_2Ga_4	✓	✗	✗
mp-13449	$YbGa_2$	Yb_1Ga_2	✓	✗	✗
mp-2451	YAl_3	Y_3Al_9	✓	✗	✗
mp-1004	Nb_3Si	Nb_3Si_1	✓	✗	✗
mp-8882	GaP	Ga_2P_2	✓	✓	✓
mp-9761	ReO_3	Re_2O_6	✓	✓	✗
mp-2231	SnS	Sn_4S_4	✓	✓	✗
mp-568382	MnBi	Mn_2Bi_2	✓	✓	✗
mp-11359	Ga_2Cu	Ga_2Cu_1	✓	✓	✗
mp-1827	$SrGa_4$	Sr_2Ga_8	✗	✗	✗
mp-30746	YIr	Y_1Ir_1	✓	✓	✗
mp-2735	PaO	Pa_1O_1	✓	✗	✗
mp-1121	GaCo	Ga_1Co_1	✓	✗	✗
mp-20225	$CePb_3$	Ce_1Pb_3	✓	✓	✗
mp-1231	Cr_3Ga	Cr_6Ga_2	✓	✓	✓
mp-2334	DyCu	Dy_1Cu_1	✓	✓	✗
mp-1169	ScCu	Sc_1Cu_1	✓	✓	✗
mp-20176	$DyPb_3$	Dy_1Pb_3	✓	✓	✗
mp-2226	DyPd	Dy_1Pd_1	✓	✓	✗
mp-788	CoTe	Co_2Te_2	✓	✗	✓

mp-20132	InHg	In_3Hg_3	✗	✗	✗
mp-13452	$BePd_2$	Be_2Pd_4	✗	✗	✗
mp-1995	PrC_2	Pr_2C_4	✗	✗	✗
mp-24658	SmH_2	Sm_4H_8	✓	✗	✗
mp-30501	Ti_2Cd	Ti_4Cd_2	✗	✗	✗
mp-30789	U_2Mo	U_4Mo_2	✗	✗	✗
mp-13181	LaF_3	La_4F_{12}	✓	✗	✗
mp-2731	$TiGa_3$	Ti_2Ga_6	✓	✗	✗
mp-454	$NaGa_4$	Na_2Ga_8	✗	✗	✗
mp-1071835	$DyCu_2$	Dy_4Cu_8	✗	✗	✗
mp-861	Hf_2Ni	Hf_8Ni_4	✗	✗	✗
mp-2129	$NdGe_2$	Nd_4Ge_8	✗	✗	✗
mp-2387	Th_2Zn	Th_8Zn_4	✗	✗	✗
mp-11251	Mg_3Au	Mg_6Au_2	✗	✗	✗
mp-30682	ZrGa	Zr_8Ga_8	✗	✗	✗
mp-11449	$HfMn_2$	Hf_4Mn_8	✗	✗	✗
mp-640079	Mn_3Au	Mn_9Au_3	✗	✗	✗
mp-1102936	Ta_2Fe	Ta_8Fe_4	✗	✗	✗
mp-1104286	Ge_4Rh	$Ge_{12}Rh_3$	✗	✗	✗
mp-1106395	Pr_3Ir	$Pr_{12}Ir_4$	✗	✗	✗
mp-1105958	Yb_3Co	$Yb_{12}Co_4$	✗	✗	✗
mp-19183	Li_2NiO_2	$Li_2Ni_1O_2$	✓	✗	✗
mp-19227	$YMnO_3$	$Y_2Mn_2O_6$	✓	✗	✗
mp-29241	Ca_3SnO	$Ca_3Sn_1O_1$	✓	✓	✗
mp-20237	CoNiSn	$Co_2Ni_2Sn_2$	✓	✓	✗
mp-11396	$NdGa_2Ni$	$Nd_2Ga_4Ni_2$	✗	✗	✗
mp-9570	$CaCd_2P_2$	$Ca_1Cd_2P_2$	✓	✗	✗
mp-30493	$ZnCdPt_2$	$Zn_1Cd_1Pt_2$	✓	✓	✗
mp-1106349	$SmPd_3S_4$	$Sm_2Pd_6S_8$	✓	✓	✓
mp-27450	$KCuCl_3$	$K_4Cu_4Cl_{12}$	✗	✗	✗
mp-11390	LiGaSi	$Li_4Ga_4Si_4$	✓	✗	✗
mp-4552	$ErInCu_2$	$Er_4In_4Cu_8$	✓	✗	✗

mp-20730	$HfCo_2Sn$	$Hf_4Co_8Sn_4$	✓	✗	✗
mp-21211	$InFeCo_2$	$In_4Fe_4Co_8$	✓	✗	✗
mp-4326	$KErS_2$	$K_3Er_3S_6$	✓	✗	✗
mp-30591	Li_2CuSn	$Li_8Cu_4Sn_4$	✓	✗	✗
mp-30648	$LiMg_2Ga$	$Li_4Mg_8Ga_4$	✓	✗	✗
mp-4972	$LuInCu_2$	$Lu_4In_4Cu_8$	✓	✗	✗
mp-5181	$LuSnPd_2$	$Lu_4Sn_4Pd_8$	✓	✗	✗
mp-20389	Na_2CdPb	$Na_8Cd_4Pb_4$	✓	✗	✗
mp-30580	$SrGaCu_2$	$Sr_3Ga_3Cu_6$	✗	✗	✗
mp-30555	$TaGaCo_2$	$Ta_4Ga_4Co_8$	✓	✗	✗
mp-24012	HoHSe	$Ho_1H_1Se_1$	✓	✗	✗
mp-5452	$CeCu_2Si_2$	$Ce_2Cu_4Si_4$	✗	✗	✗
mp-3147	$ErSi_2Au_2$	$Er_2Si_4Au_4$	✗	✗	✗
mp-13405	$LuMn_2Ge_2$	$Lu_2Mn_4Ge_4$	✗	✗	✗
mp-30805	$SrNiSn_3$	$Sr_2Ni_2Sn_6$	✗	✗	✗
mp-12743	$CrTe_4Au$	$Cr_1Te_4Au_1$	✗	✗	✗
mp-7524	NbPSe	$Nb_4P_4Se_4$	✗	✗	✗
mp-11435	$ErTi_2Ga_4$	$Er_2Ti_4Ga_8$	✗	✗	✗
mp-7095	$CeCoSi_2$	$Ce_4Co_4Si_8$	✗	✗	✗
mp-5615	CaAgAs	$Ca_3Ag_3As_3$	✗	✗	✗
mp-30733	HoSnPt	$Ho_3Sn_3Pt_3$	✗	✗	✗
mp-16747	LuAgPb	$Lu_3Ag_3Pb_3$	✗	✗	✗
mp-13171	YMgCu	$Y_3Mg_3Cu_3$	✗	✗	✗
mp-7550	$CeNbO_4$	$Ce_2Nb_2O_8$	✗	✗	✗
mp-12931	$SmTaO_4$	$Sm_2Ta_2O_8$	✗	✗	✗
mp-7913	$BeSiN_2$	$Be_4Si_4N_8$	✓	✗	✗
mp-1106406	Ce_2SnS_5	$Ce_4Sn_2S_{10}$	✗	✗	✗
mp-1106117	La_5AgPb_3	$La_{10}Ag_2Pb_6$	✗	✗	✗
mp-1106245	Zr_5AlSb_3	$Zr_{10}Al_2Sb_6$	✗	✗	✗
mp-557997	$CaSeO_3$	$Ca_4Se_4O_{12}$	✗	✗	✗
mp-19140	K_3MnO_4	$K_6Mn_2O_8$	✗	✗	✗
mp-6258	$CeCr_2Si_2C$	$Ce_1Cr_2Si_2C_1$	✓	✗	✗

mp-726253	$RbLi_3S_2O_9$	$Rb_1Li_3S_2O_9$	✗	✗	✗
mp-23126	KAs_4IO_6	$K_1As_4I_1O_6$	✗	✗	✗
mp-12515	LaZnSbO	$La_2Zn_2Sb_2O_2$	✓	✗	✗
mp-1071272	$CeAl_2BRu_2$	$Ce_1Al_2B_1Ru_2$	✓	✗	✗
mp-1213726	CrFeCoSi	$Cr_1Fe_1Co_1Si_1$	✓	✓	✗
mp-552674	ZrTaNO	$Zr_1Ta_1N_1O_1$	✓	✓	✗
mp-7554	LiMgSnAu	$Li_4Mg_4Sn_4Au_4$	✓	✗	✗
mp-11806	LiMgSnPt	$Li_4Mg_4Sn_4Pt_4$	✓	✗	✗
mp-6794	LaB_2Rh_2C	$La_2B_4Rh_4C_2$	✓	✗	✗
mp-6140	$PrNi_2B_2C$	$Pr_2Ni_4B_4C_2$	✗	✗	✗
mp-545788	Ba_3ZnN_2O	$Ba_3Zn_1N_2O_1$	✗	✗	✗
mp-13287	BaCuTeF	$Ba_2Cu_2Te_2F_2$	✗	✗	✗
mp-1221640	MnAlCuPd	$Mn_1Al_1Cu_1Pd_1$	✓	✗	✗
mp-546790	LaCuTeO	$La_2Cu_2Te_2O_2$	✓	✗	✗
mp-18745	LaMnSbO	$La_2Mn_2Sb_2O_2$	✗	✗	✗
mp-20349	SmFeAsO	$Sm_2Fe_2As_2O_2$	✗	✗	✗
mp-12444	SrCuSF	$Sr_2Cu_2S_2F_2$	✗	✗	✗
mp-20807	SrFeAsF	$Sr_2Fe_2As_2F_2$	✗	✗	✗
mp-19118	$BaNd_2CoO_5$	$Ba_2Nd_4Co_2O_{10}$	✗	✗	✗
mp-23127	Ba_2BiSbO_6	$Ba_4Bi_2Sb_2O_{12}$	✗	✗	✗
mp-21348	Ba_2EuTaO_6	$Ba_4Eu_2Ta_2O_{12}$	✗	✗	✗
mp-19274	$BaPrMn_2O_6$	$Ba_1Pr_1Mn_2O_6$	✗	✗	✗
mp-1095437	$LuSeO_3F$	$Lu_2Se_2O_6F_2$	✗	✗	✗
mp-1106310	Cu_2SiHgS_4	$Cu_4Si_2Hg_2S_8$	✗	✗	✗
mp-1105386	YbH_3CN_3	$Yb_2H_6C_2N_6$	✗	✗	✗
mp-651268	$Fe_2Cu_6SnS_8$	$Fe_2Cu_6Sn_1S_8$	✗	✗	✗
mp-1105110	$Sr_2Bi_2Se_3O_2$	$Sr_4Bi_4Se_6O_4$	✗	✗	✗
mp-1105674	$Mg_2BeB_2Ir_5$	$Mg_4Be_2B_4Ir_{10}$	✗	✗	✗
mp-13383	$ScAgP_2Se_6$	$Sc_2Ag_2P_4Se_{12}$	✗	✗	✗

References

(S1) Cheng, G.; Gong, X.-G.; Yin, W.-J. Crystal structure prediction by combining graph network and optimization algorithm. *Nature Communications* **2022**, *13*, 1492.

(S2) Miranda, L. J. PySwarms: a research toolkit for Particle Swarm Optimization in Python. *Journal of Open Source Software* **2018**, *3*, 433.

(S3) Omee, S. S.; Wei, L.; Hu, M.; Hu, J. Crystal structure prediction using neural network potential and age-fitness Pareto genetic algorithm. *Journal of Materials Informatics* **2024**,

(S4) Blank, J.; Deb, K. Pymoo: Multi-objective optimization in python. *IEEE Access* **2020.** https://doi.org/10.1109/ACCESS.2020.2990567, *8*, 89497–89509.

(S5) Deb, K.; Jain, H. An evolutionary many-objective optimization algorithm using reference-point-based nondominated sorting approach, part I: solving problems with box constraints. *IEEE Transactions on Evolutionary Computation* **2013**, *18*, 577–601.

(S6) Jain, H.; Deb, K. An evolutionary many-objective optimization algorithm using reference-point based nondominated sorting approach, part II: Handling constraints and extending to an adaptive approach. *IEEE Transactions on Evolutionary Computation* **2013.** https://doi.org/10.1109/TEVC.2013.2281534, *18*, 602–622.

(S7) Deb, K.; Sindhya, K.; Okabe, T. Self-adaptive simulated binary crossover for real-parameter optimization. Proceedings of the 9th Annual Conference on Genetic and Evolutionary Computation. 2007; pp 1187–1194.

(S8) Schmidt, M. D.; Lipson, H. Age-fitness pareto optimization. *Proceedings of the 12th Annual Conference on Genetic and Evolutionary Computation.* 2010; pp 543–544.

(S9) Schmidt, M. D.; Lipson, H. Age-fitness pareto optimization. *Genetic Programming Theory and Practice VIII* **2010.** https://doi.org/10.1007/978-1-4419-7747-2_8, *8*, 129.

(S10) Fredericks, S.; Parrish, K.; Sayre, D.; Zhu, Q. PyXtal: A Python library for crystal structure generation and symmetry analysis. *Computer Physics Communications* **2021**, *261*, 107810.

(S11) Chen, C.; Ong, S. P. A universal graph deep learning interatomic potential for the periodic table. *Nature Computational Science* **2022**, *2*, 718–728.

(S12) Deng, B.; Zhong, P.; Jun, K.; Riebesell, J.; Han, K.; Bartel, C. J.; Ceder, G. CHGNet as a pretrained universal neural network potential for charge-informed atomistic modelling. *Nature Machine Intelligence* **2023**, *5*, 1031–1041.

(S13) Park, Y.; Kim, J.; Hwang, S.; Han, S. Scalable parallel algorithm for graph neural network interatomic potentials in molecular dynamics simulations. *Journal of Chemical Theory and Computation* **2024**, *20*, 4857–4868.

(S14) Fu, X.; Wood, B. M.; Barroso-Luque, L.; Levine, D. S.; Gao, M.; Dzamba, M.; Zitnick, C. L. Learning Smooth and Expressive Interatomic Potentials for Physical Property Prediction. *arXiv preprint arXiv:2502.12147* **2025. Preprint at:** `https://doi.org/10.48550/arXiv.2502.12147`,

(S15) Ong, S. P.; Richards, W. D.; Jain, A.; Hautier, G.; Kocher, M.; Cholia, S.; Gunter, D.; Chevrier, V. L.; Persson, K. A.; Ceder, G. Python Materials Genomics (pymatgen): A robust, open-source python library for materials analysis. *Computational Materials Science* **2013**, *68*, 314–319.

(S16) Momma, K.; Izumi, F. VESTA: a three-dimensional visualization system for electronic and structural analysis. *Journal of Applied Crystallography* **2008**, *41*, 653–658.

(S17) Wei, L.; Li, Q.; Omee, S. S.; Hu, J. Towards quantitative evaluation of crystal structure prediction performance. *Computational Materials Science* **2024**, *235*, 112802.

(S18) Rubner, Y.; Tomasi, C.; Guibas, L. J. The earth mover's distance as a metric for image retrieval. *International journal of computer vision* **2000**, *40*, 99–121.

(S19) Zimmermann, N. E.; Jain, A. Local structure order parameters and site fingerprints for quantification of coordination environment and crystal structure similarity. *RSC Advances* **2020**, *10*, 6063–6081.
